# Are intentions to change, policy awareness, or health knowledge related to changes in dietary intake following a sugar-sweetened beverage tax in South Africa? A before-and-after study

**DOI:** 10.1186/s12966-022-01370-5

**Published:** 2022-10-28

**Authors:** Michael Essman, Catherine Zimmer, Francesca Dillman Carpentier, Elizabeth C. Swart, Lindsey Smith Taillie

**Affiliations:** 1 Carolina Population Center and Gillings School of Global Public Health, Department of Nutrition Chapel Hill, University of North Carolina at Chapel Hill, Chapel Hill, NC United States of America; 2 Odum Institute for Research in Social Science, University of North Carolina at Chapel Hill, Chapel Hill, NC United States of America; 3 University of North Carolina at Chapel Hill, Hussman School of Journalism and Media, Chapel Hill, NC United States of America; 4 Department of Dietetics and Nutrition, University of the Western Cape, Robert Sobukwe Rd, Bellville, Cape Town, South Africa

**Keywords:** Obesity prevention, Sugary drinks, Tax, Policy evaluation, Behavior change

## Abstract

**Background:**

In April 2018, South Africa implemented the Health Promotion Levy (HPL), one of the first sugar-sweetened beverage (SSB) taxes to be based on each gram of sugar (beyond 4 g/100mL). The objectives of this study were to examine whether the psychological constructs tax awareness, SSB knowledge, SSB risk perception, and intentions to reduce SSB intake were associated with taxed beverage intake, whether they changed from pre- to post-tax, and whether they modified the effect of the HPL.

**Methods:**

We collected single day 24-hour dietary recalls surveyed from repeat cross-sectional surveys of adults aged 18–39 years in Langa, South Africa. Participants were recruited in February-March 2018 (pre-tax, N = 2,481) and February-March 2019 (post-tax, N = 2,507) using door-to-door sampling. Surveys measured tax awareness, SSB knowledge, SSB risk perception, and intention to reduce SSB intake. SSB intake was estimated using a two-part model. To examine changes over time, logistic regression models were used for binary outcomes (tax awareness and intention to reduce SSB consumption) and linear regression models for continuous outcomes (SSB knowledge SSB risk perceptions). Effect modification was tested using interaction terms for each psychological construct with time.

**Results:**

No constructs were associated with SSB intake at baseline. At post-tax, the predicted probability to consume taxed beverages was 33.5% (95% CI 28.5–38.5%) for those who expressed an intention to reduce SSB intake compared to 45.9% (95% CI 43.7–48.1%) for those who did not. Among consumers, intending to reduce SSB intake was associated with 55 (95% CI 28 to 82) kcal/capita/day less SSBs consumed. Tax awareness, SSB knowledge, and SSB risk perception increased by a small amount from pre- to post-tax. Intentions to reduce SSB intake was lower in the post-tax period. The tax effect on SSB intake was modified by SSB knowledge and intention to reduce SSB intake, with higher levels of each associated with lower SSB intake.

**Conclusion:**

After the South African SSB tax was implemented, SSB knowledge and risk perception increased slightly, tax awareness remained low, and only SSB knowledge and behavioral intention to change were significantly associated with taxed beverage intake among participants recruited from a low-income South African township.

**Supplementary Information:**

The online version contains supplementary material available at 10.1186/s12966-022-01370-5.

## Introduction

Sugar-sweetened beverage (SSB) consumption is linked to obesity [1, 2] and other non-communicable diseases [3, 4] and is increasing rapidly in low- and middle-income countries [5]. In response to rising SSB purchases, obesity, and type II diabetes incidence [6–8], South Africa became the first country on the sub-Saharan African continent to implement a sugary beverage tax, called the Health Promotion Levy (HPL), in April 2018 [9]. The HPL has a unique structure that applies a fixed 2.1 cent tax for each additional gram of sugar (both intrinsic and added) above a 4 g/100 ml threshold [9]. The combination of the threshold and the increasing taxation for each gram of added sugar has not been tried nor tested elsewhere. The goal of this tax design is not only to increase prices, thereby reducing consumer purchases of SSBs, but also to spur beverage reformulation by industry [10]. A secondary objective was to raise revenue for the national budget [11], which could in principle help support a financially strained health care system [12], although specific health allocations were not agreed.

The clearest examples of sugar-based national SSB tax structures are from the United Kingdom’s threshold-based multi-tiered SSB levy [13], South Africa’s HPL, and Mauritius [14]. Evaluations have found reductions in taxed beverage consumption post-tax, leading to fewer calories and grams of sugar consumed per capita. In the United Kingdom, the greatest changes in sugar content of beverage purchases were due to reformulation, with a reduction of 30% but only 4% without accounting for reformulation [15]. In Mauritius, the young male consumers of SSBs fell 11% [14]. In South Africa, taxed beverage purchases decreased 33% in lower and middle income and 20% in higher income populations [16]. Another study using dietary intake data collected from a low income, high consuming South African township separated the effects of behavioral change from reformulation, finding a 24% reduction in taxed beverage caloric intake due to behavioral change and an additional 8% reduction due to reformulation [17].

In light of these results, a remaining question is what drives these behavioral changes. Price increases lead to reduced SSB purchases with varying effects by age, income and country [18, 19]. However, behavior change is complex and likely related to more than SSB price changes, including psychological factors that influence consumer responses to SSB taxes. Additionally, national SSB taxes are often accompanied by mass media campaigns to promote the tax and inform the public about the purpose of the tax to reduce the disease burdens of obesity and diabetes [20, 21]. Media campaigns about the health harms of SSBs have been shown to increase tax awareness, increase perceived risk about the health harms of SSBs, and increase behavioral intentions to reduce SSB consumption [20–24]. However, to our knowledge, no studies have linked these psychological constructs with changes in dietary intake, the ultimate goal of SSB tax policy. Finally, although knowledge about the health harms of SSBs is inversely associated with SSB intake [25], it is unknown whether changes in knowledge or behavioral intentions may modify the effects of SSB taxes. If changes in knowledge modify the effects of SSB taxes, then information campaigns to increase SSB knowledge may complement future SSB tax policies, leading to even greater reductions in SSB intake.

The objectives of this study were to examine whether the psychological constructs tax awareness, intentions to reduce SSB intake, SSB knowledge, and SSB risk perception were associated with taxed beverage intake, whether their levels changed from pre- to post-tax, and whether they modified the effect of the tax on taxed beverage intake.

## Methods

This study was part of a student dissertation and not registered in a public repository. However, the original proposal of the study design and analytical plan from the dissertation proposal can be found here: https://cdr.lib.unc.edu/concern/dissertations/kd17d2883. This study is reported as per the Strengthening the Reporting of Observational Studies in Epidemiology (STROBE) guideline (Supplementary Material 1. STROBE-nut checklist).

### Data sources and measures

#### Participants

Data were collected using cross-sectional surveys of adults aged 18–39 years living in the lower income Langa township near Cape Town, South Africa. Our study population in the Langa township was selected because it contains a large number of adults who are high consumers of SSBs and is primarily a low income population, and both of these characteristics have been associated with greater reductions in unhealthy food or beverage purchases following a tax [26–28]. Another reason Langa was selected as a suitable township for the study was because some townships do not have residents who remain for long periods of time, whereas Langa is more likely to have people living there permanently. This stability made Langa suitable to do this before-and-after comparison study to have comparable populations at both time points. At last count, Langa had 17,402 households and 52,401 inhabitants (50.4% female), of which 99.1% were of Black African race [29].

Participants were recruited using a door-to-door sampling method of all identifiable households in Langa until the target sample size of approximately 2,500 households was achieved at each wave/collection. The household questionnaires were conducted digitally, which were linked to geolocations to ensure that we sampled from all areas of the township. These geocodes were used to create a map, which could be examined for coverage of the entire township (Supplementary Material 2). The area with single dots are brick houses, and the area with dense dots includes informal housing and flats. Open spaces are in general communal spaces such as playgrounds, churches, schools, police, health clinics or shops (Supplementary Material 2). Participants received a supermarket voucher worth R30 (USD$2.19) after participating.

Because the survey was designed to capture adults’ SSB intake, the only eligibility criterion was being between 18 and 39 years of age. Every household was approached to ascertain if they met the age requirement and if they were willing to participate. Only one diet assessment and one knowledge questionnaire were completed for a single individual within a given household. Each household was assigned an ascending household number as they were entered into the online database, and this household number was used to select which eligible participant to survey when more than one was present in households. Eligible participants in each household were numbered in the order in which they confirmed their age eligibility. If there were two qualifying participants present in the household, the first qualifying participant was selected if the household number in the survey was an uneven number, and the second participant was selected if the household number was an even number. If three or more qualifying participants were present in the household, a random numbers list was used to select the respondent.

Respondents were disproportionately female as females were more likely to be at home compared to males when sampling participants during daytime hours (Table 1).

**Table 1:** Sociodemographic information for participants with complete survey data

Variable​	Pre-tax (n = 2,094)	Post-tax (n = 2,316)	p value*
	%	%	
Male	35.1	34.9	0.897
Female	65​0.0	65​0.1	
Diet surveyed on weekday	82.9	83.9	0.264
LSM category^1^			< 0.001
LSM 3	1.3	1.7	
LSM 4	14.8	19.8	
LSM 5	39.0	51.5	
LSM 6	39.2	26.3	
Missing/incomplete data	5.7	0.7	
	Mean (SD)	Mean (SD)	0.207
Age	27.9 (6.0)	27.8 (6.2)	

^1^South African Living Standards Measure (LSM) [32]

*p value for difference calculated using Fisher’s exact test and means with two-tailed t-test

Data were collected in a pre-tax survey in February-March 2018, two months before the tax implementation in April 2018 (N = 2,481) and a post-tax survey 12 months later in February-March 2019 (N = 2,507). Among the households that were accessible and had eligible participants at home, the refusal rate was 11.9% in the pre-tax period and 4.2% in the post-tax period. Households that were not accessible or had no eligible participants at home were not counted as refusals. Diet records reporting less than 400 daily kcal were deemed unreliable for having an implausibly low daily energy intake and were dropped, totaling 22 diet records in the pre-tax group (0.9%) and 18 diet records in the post-tax group (0.7%), leaving 2,459 participants in pre-tax and 2,489 in post-tax groups (Table 1). Our final analytic sample contained only variables with complete survey data totaling 2,094 in the pre-tax group and 2,316 in the post-tax group (Table 1). 293 (12.7%) participants in the post-tax period reported they were also included in the pre-tax survey.

#### Measuring dietary intake

For the diet assessment, 24-hour diet recalls were conducted by interviewers with nutrition training using the multiple pass approach—including uninterrupted recall of foods and beverages consumed the previous day, following by detailed prompting for individual foods and portion sizes—to enhance completeness. Langa has a large number of Xhosa speakers, and interviewers were fluent in both Xhosa and English. Interviews were conducted in the language with which participants were most comfortable. Diet recalls and questionnaires were conducted aloud with all participants and recorded by interviewers. Participants reported what foods and drinks were eaten, how foods and beverages were prepared, whether anything was added, and the quantity consumed.

#### Linking dietary data to beverage categories

Data from 24-hour dietary recalls were linked to composite nutritional records for beverages based on the current food supply and consumer purchases. First, nutrition facts panel (NFP) data were collected from South African grocery stores during the same time period (February-March 2018) as diet intake data. The NFP data were linked to a database of beverage codes, allowing linkages between NFP data and dietary intake data. Each beverage code was given an average nutrient profile, weighted by household purchase data from Kantar World Panel 2018, a panel dataset of household packaged food and beverage purchases. Beverage codes were then categorized into taxed and untaxed categories based on the linked average nutrient profile. Beverage taxation status was determined by a two-step process: (1) whether the product category is taxable, as 100% fruit juice and unsweetened milks are exempt from the tax, and (2) among all other beverages that are taxable, those with a total sugar concentration greater than 4 g/100ml are classified as taxed and those with 4 g/100ml or less are untaxed. We considered all tax-exempt and < 4 g sugar/100ml beverages as untaxed. The same food composition tables were used for our entire study period, which restricts all effects to behavioral change only and not reformulation. All beverages were assigned to either taxed or untaxed categories.

#### Measuring psychological constructs

After completion of the dietary intake assessment, participants completed a knowledge and attitudes questionnaire modeled after previous work that surveyed SSB-related knowledge, attitudes and behaviours, including a study conducted in South Africa [30, 31]. To measure the knowledge construct, participants were asked, “Is the following beverage sugary?” for each of the 11 beverage types listed in Table 2. Next, after participants were given a definition of SSBs, they were asked to what degree SSB consumption increases the risk of selected chronic diseases and risk factors listed as the Risk Perception Construct in Table 2. For SSB knowledge and risk perception, the average scores for risk perception and SSB knowledge was calculated at each time period, with partial credit also awarded. For risk perception, 0 points were given if the participant perceived no risk; 0.333 points were given if the risk was perceived as “a little”; 0.666 points were given if the risk was perceived as “somewhat”; and 1 point was given if the risk was perceived as “a lot.” For SSB knowledge, 0 points were awarded if the response was incorrect (e.g. a response of “not sugary” when the correct answer was “sugary”); 0.5 points were awarded if the response was partially correct (e.g. a response of “somewhat sugary” when the correct answer was “sugary”); and 1 point was awarded if the response was correct. Partial credit was not awarded to responses of “somewhat sugary” for beverages that contained no added sugars, as this is not merely a difference of degree but incorrect altogether. We asked whether beverages were sugary, not whether they were taxed, because we did not want to assume participants’ knowledge of the tax and queried for tax awareness in another question. To assess perception of the tax, participants were asked whether they were aware of the SSB tax (yes/no) and whether they planned to reduce SSB consumption as a result of the tax (yes/no).

**Table 2:** Psychological constructs measured in the present study

Construct	Definition	Question Items	Response type
Risk Perception	Expressed beliefs about potential health harms of consuming SSBs*	**Question: To the best of your knowledge, does consumption of sugary drinks increase the suffering from…?**	Categorical 1: Not at all 2: A little 3: Somewhat 4: A lot 5: Not sure (coded missing)
		Q1: Diabetes	
		Q2: Blood Pressure	
		Q3: Obesity	
		Q4: Cavities	
Knowledge	Ability to correctly identify sugary beverages from a list	**Question: Is the following beverage sugary?**	Categorical 1: Not sugary 2: Somewhat sugary 3: Sugary 4: Do not know (coded missing)
		Q1: Flavored bottled water	
		Q2: 100% fruit juice	
		Q3: Nectars or canned juice that contain fruit (e.g. Tropicana)	
		Q4: Milk (sweetened and flavored) (e.g. Nesquick, Steristumpie)	
		Q5: Soda or soft drinks (e.g. Coca-Cola, Sprite, ginger beer)	
		Q6: Sweetened Iced tea (BOS, Lipton ice tea, Fuze)	
		Q7: Coffee/tea with sugar (including cappuccino, frapuccino)	
		Q8: Energy drinks (Red Bull, Monster, Dragon)	
		Q9: Sports drinks (e.g. Energade, Powerade, Lucozade)	
		Q10: Powdered drinks (e.g. Game)	
		Q11: Cordials and concentrates (e.g. Oros)	
Tax Awareness	Aware of the South African SSB* tax	Question: “Are you aware of the new Health Promotion Levy (also called Sugary Beverage Tax)?”	Binary (yes/no)
Intention to reduce SSBs*	Expressed an intention to reduce SSB* consumption as a result of the tax	Question: “The Government has approved a new tax on sugary sweetened beverages which will come into effect on 1 April 2018. If this tax will result in an increase in price of about R2 for 2 liters of sugary beverages, how likely will it have the following effect on your purchasing intentions?” Response: “I will cut back on my sweetened beverage consumption (yes/no)”	Binary (yes/no)

* SSB = sugar-sweetened beverage

#### Main outcome and covariates

The main outcome for analysis questions 1 and 3 below was calories from taxed beverage intake. This outcome was selected because calorie intake is related to health outcomes. To examine changes over time for question 2, SSB knowledge, risk perception, tax awareness, and intention to reduce SSB intake were each used as outcomes. Covariates for adjusting all analyses included age (continuous, range 18–39), sex, and weekday versus weekend of intake (binary). Socioeconomic status was assigned using the South African Audience Research Foundation’s Living Standards Measure (LSM) as a categorical variable [32]. LSM is designed to segment households into categories ranging from 1 to 10, but our sample only includes participants in the lower and middle part of the range, from 3 to 6 due to the income profile of persons living in Langa. Adjusting for LSM changed our calculation of daily energy intake from taxed beverages by less than a 1 kcal per capita per day. We therefore concluded that our results were not confounded by LSM and did not control for it in our final models due to the amount of missing data (16%) at baseline.

### Analytical Approach

This analysis sought to answer three research questions, including whether the psychological constructs tax awareness, intentions to reduce SSB intake, SSB knowledge, and SSB risk perception were associated with taxed beverage intake, whether their levels changed from pre- to post-tax, and whether they modified the effect of the tax on taxed beverage intake.

To examine whether psychological constructs were associated with taxed beverage intake, we estimated beverage consumption using a two-part model [33] in Stata 16 [34] to account for zero values of taxed beverage intake due to non-consumers, with a probit model for the first part (likelihood of consumption), and conditional on consumption, a generalized linear model with log-link for the second part [35]. This type of model links the probability to consume with the amount consumed [36]. We used taxed beverage energy intake (kcal) as the dependent variable and tax awareness, risk perception, knowledge, and intention to reduce SSB consumption as independent variables. Models were adjusted in both steps for age, sex, and weekday versus weekend of intake. If the psychological constructs were associated with taxed beverage intake at either time period, we estimated predicted probability to consume and predicted taxed beverage intake among consumers to compare differences in intakes between groups.

To examine whether levels of psychological constructs changed from pre- to post-tax, we examined whether the population means for our potential modifiers were equal at both time points. We used a logistic regression model to examine changes in binary outcomes (tax awareness and intention to reduce SSB consumption) and linear regression models for continuous outcomes (SSB knowledge SSB risk perceptions).

To examine whether psychological constructs modified the effect of the tax on taxed beverage intake, we used the same two-part model as question 1, while also testing interaction terms for each psychological construct with time. A statistically significant model coefficient for an interaction term (p < 0.05) suggests the latent variable modifies the effect of the tax over time. Adjusted predictions for taxed beverage intakes are reported for those aware and unaware of the tax, intending and not intending to change their intakes, and increasing levels of SSB knowledge and risk perception [37].

## Results

### Sociodemographic characteristics

Study population characteristics are presented in Table 1. From pre-tax to post-tax, the percent of respondents in LSM categories 4 and 5 increased (p < 0.001), and the percent of respondents in the highest LSM category 6 decreased (p < 0.001). There were no significant differences between the two time points for other sociodemographic characteristics.

#### Summary Statistics for the Sample

Descriptive statistics for the sample are presented in Table 3. Mean taxed beverage intakes were greater for males compared to females in both the pre-tax period (128 kcal/capita/day, SD 154 compared to 128 kcal/capita/day, SD 140, respectively) and the post-tax period (102 kcal/capita/day, SD 147 compared to 85 kcal/capita/day, SD 128, respectively) (Table 3). There was an inverse relationship between age and taxed beverage intake, and 18–24 year olds had the highest intakes in both the pre- (135 kcal/capita/day, SD 152) and post-tax (102 kcal/capita/day, SD 143) periods (Table 3). Comparing by socioeconomic status, the greatest taxed beverage intakes were among the lowest LSM category in both the pre- (153 kcal/capita/day, SD 145) and post-tax (106 kcal/capita/day, SD 134) periods (Table 3).

**Table 3:** Mean taxed beverage intake by demographic groups for pre- and post-tax periods

Variable​	Pre-tax	Post-tax
	(n = 2,009)	(n = 2,309)
	Mean (SD)	Mean (SD)
Sex		
Male	128 (154)	102 (147)
Female	118 (140)	85 (128)
Age		
18–24	135 (152)	102 (143)
25–29	127 (143)	92 (128)
30–34	114 (146)	85 (140)
35–39	99 (128)	77 (122)
LSM category^1^		
LSM 3	153 (145)	106 (134)
LSM 4	122 (149)	86 (144)
LSM 5	120 (137)	92 (138)
LSM 6	121 (150)	93 (123)
Missing	128 (147)	61 (102)

^1^South African Living Standards Measure (LSM) [32]

#### Regression results

1. *Are SSB knowledge and SSB risk perception, tax awareness, or intentions to reduce SSB consumption associated with taxed beverage intake?*

In models examining taxed beverage intake at baseline, there was no significant association between tax awareness, SSB knowledge, SSB risk perception, or intention to reduce SSB intake and odds of consuming taxed beverages or the consumption-day amount. In the post-tax period, the intention to reduce SSB intake was significantly associated with 0.59 times the odds (95% CI 0.46 to 0.75) of consuming taxed beverages compared to those who did not intend to reduce SSB intake. The predicted probability to consume taxed beverages was 33.5% (95% CI 28.5% − 38.5%) for those who expressed an intention to reduce SSB intake compared to 45.9% (95% CI 43.7–48.1%) for those who did not. Participants who intended to reduce SSB intake who were also consumers of taxed beverages consumed 55 (95% CI 28 to 82) kcal/capita/day less than consumers who did not express intention to change.

2) *Did mean SSB knowledge, SSB risk perceptions, tax awareness, or intention to reduce SSB consumption change from pre-tax to post-tax?*

In models examining changes in the psychological constructs before and after the tax, the adjusted percentage reporting they were aware of the tax increased from 12.8 to 16.0% of the sample (p = 0.003). SSB knowledge increased slightly, from 70.2% correct pre-tax to 72.3% correct post-tax (p < 0.0001). Risk perception increased slightly, from 76.0% of risks perceived pre-tax to 79.6% of risks perceived post-tax (p < 0.0001). The adjusted percentage reporting the intention to reduce SSB consumption decreased significantly (p < 0.001) from 43.1% pre-tax to 15.0% post-tax.

3) *Do SSB knowledge and SSB risk perception, tax awareness, or intentions to reduce SSB consumption modify the effect of time on taxed beverage intake?*

Finally, we tested interaction models to determine whether the relationships between the psychological constructs and the tax effect changed over time. Overall, we found the tax effect on SSB intake was modified by SSB knowledge and intention to reduce SSB intake, and the tax effect on SSB intake was not modified by tax awareness or risk perception.

For tax awareness, the interaction term was not statistically significant (p = 0.103). Changes in energy intake from pre- to post-tax were predicted to be -19 (95% CI -42 to 4) kcal/capita/day lower among those aware of the tax compared to not aware. For within-group differences, among those aware of the tax at both time periods, taxed beverage intake decreased from 131 (95% CI 113 to 148) to 83 (95% CI 70 to 97) kcal/capita/day (Table 4). Among those unaware of the tax at both time periods, taxed beverage intake decreased from 121 (95% CI 114 to 127) to 93 (95% CI 87 to 99) kcal/capita/day.

**Table 4:** Predicted estimates for taxed beverage intake pre- and post-tax implementation by tax awareness, intention to change SSB intake, and levels of SSB knowledge and risk perception

Psychological Construct	Taxed beverage intake (kcal per capita per day)	
**Awareness**	**Pre (95% CI)**	**Post (95% CI)**
Yes	130	84
	(113–147)	(71–97)
No	120	93
	(114–127)	(87–99)
**Intention to change**		
Yes	116	61
	(107–125)	(49–73)
No	125	97
	(117–133)	(91–103)
**SSB Knowledge**		
30%	107	105
	(91–123)	(87–123)
40%	110	102
	(97–123)	(88–116)
50%	113	99
	(103–123)	(89–109)
60%	117	96
	(110–124)	(89–103)
70%	120	93
	(114–126)	(87–98)
80%	124	89
	(116–131)	(83–96)
90%	128	86
	(117–138)	(78–95)
100%	131	83
	(117–146)	(72–94)
**SSB Risk Perception**		
30%	119	95
	(106–132)	(82–108)
40%	119	94
	(109–130)	(84–105)
50%	120	94
	(111–129)	(85–103)
60%	120	93
	(113–127)	(86–100)
70%	121	92
	(114–127)	(87–98)
80%	121	92
	(115–127)	(86–97)
90%	121	91
	(114–128)	(85–97)
100%	122	90
	(113–130)	(83–98)

There was a statistically significant interaction (p = 0.03) between SSB knowledge and time period on the probability to consume taxed beverages. SSB knowledge was measured using a percentage of beverage types correctly classified as sugary, with a mean of 70.3% and a standard deviation of 15.2% at baseline. Comparing the post-tax intakes to the pre-tax intakes, there were greater reductions in SSB intake with each 10-percentage point increase in knowledge questions answered correctly (Table 4). In the post-tax period, each 10% point increase in knowledge questions answered correctly was associated with an approximately 6 kcal/capita/day greater reduction in taxed beverage intake (Table 4).

There was a statistically significant interaction (p < 0.01) between intention to reduce SSB consumption and time period on predicted taxed beverage intake. While there was a reduction in adjusted mean SSB calories for both groups, reductions in energy intake from pre- to post-tax were predicted to be 27 (95% CI 9 to 46) kcal/capita/day greater among those expressing an intention to reduce SSB intake compared to no intention to reduce SSB intake. For within-group differences, among those who intended to reduce SSB consumption at both time periods, taxed beverage intake decreased from 116 (95% CI 107 to 125) to 61 (95% CI 49 to 73) kcal/capita/day (Table 4). Among those who did not intend to reduce SSB consumption at both time periods, taxed beverage intake decreased from 125 (95% CI 117 to 133) to 97 (95% CI 91 to 103) kcal/capita/day (Table 4).

## Discussion

This study examined whether SSB knowledge, risk perception, tax awareness, and intentions to reduce SSB intake were associated with changes in taxed beverage intake in a low income township following South Africa’s SSB tax. We also tested whether these psychological constructs changed over time or modified the effects of the tax on taxed beverage intake. In the pre-tax period, none of our psychological measures were associated with taxed beverage intake. In the post-tax period, both SSB knowledge and intention to reduce SSB intake were significantly associated with taxed beverage intake. Intention to reduce SSB intake was associated with lower probability to consume taxed beverages and a lower intake among consumers compared to no intention to change, and there was a statistically significant inverse relationship between SSB knowledge and taxed beverage intake in the post-tax period. Tax awareness, SSB knowledge, and SSB risk perception increased by small amounts after the tax, and intention to reduce SSB intake decreased after the tax. When testing for modification of the tax effect, only intention to reduce SSB intake and SSB knowledge modified the association between policy implementation and dietary intake, with greater post-tax reductions in taxed beverage intake among those reporting an intention to change compared to those who did not.

Previous work from the same study sample in Langa, South Africa found reductions in taxed beverage intake due to behavioral change (i.e., an average decrease of 24% in calories/capita/day of taxed beverage intake) [17], and this study sought to examine the potential components of this behavioral change. In light of our results, these reductions are unlikely to be explained by changes in SSB knowledge, risk perception, and awareness of the HPL. Our results agree with those from Murukutla and colleagues, who found awareness of an SSB tax supporting media campaign was not associated with behavioral change, suggesting tax awareness is not sufficient to spur behavioral change [21]. A more likely explanation could be the price sensitivity of lower income individuals. Our study included LSM categories ranging from 3 to 6 on the 10-point LSM scale used in South Africa to estimate household economic status [38]. LSM categories 1–4 have the least access to wealth, typically with primary school and some high school education, and an approximate household income of 85–200 USD per month [38]. LSM categories 5–7 have moderate access to wealth, typically with high school and some higher education, and an approximate household income of 260–700 USD per month [38], although the highest our sample included was 6 in the middle of this range. Indeed, qualitative research from focus groups of literate South Africans adults found that price was a more commonly mentioned reason for selecting a product compared to nutrition information [39]. Research from Mexico [26] and Chile [40] found lower income individuals were more sensitive to SSB price changes. The South African HPL is approximately a 10–11% tax [16], effectively similar in magnitude to other taxes such as the 10% tax in Mexico [41] and the 11.0% tax on large containers (20 oz. bottles) in Berkeley [42].

SSB knowledge, risk perception, and tax awareness increased after the tax was implemented, but these changes were small. In the post-tax period, SSB knowledge was 2.1% points greater, and risk perception was 3.6% points greater. Such small increases suggest a need for more widespread media campaigns to further increase knowledge and risk perceptions, particularly in low income settings. Previous research found that media campaigns to prevent obesity have led to increased knowledge and concern about obesity as a health issue [43–46]. However, these studies use psychological measures as their final outcomes, and greater emphasis may be needed to link these psychological measures with dietary intake outcomes to examine the ultimate intended effects on intake. Simply implementing a tax may not be enough to change beliefs or risk perceptions about SSB consumption. More media and communication to accompany the policy that raises awareness of the tax and its purpose—particularly among the highest risk populations—followed by education efforts could improve public awareness and understanding of the policy.

Our study found small increases in tax awareness from pre- to post-tax, but awareness remained low. Other studies from South Africa have found low awareness of the tax and skepticism that it will improve health [47]. There may also be income-based disparities in tax awareness. An analysis of a media campaign to raise awareness of the link between SSBs and chronic diseases in South Africa called *Are You Drinking Yourself Sick?* found lower awareness of the campaign among lower socioeconomic status adults [21]. Given the findings of the present study, tax awareness may not be enough to change behavior, and strategies to increase intentions to change as well as SSB knowledge could benefit lower income populations. However, given the small effects of increasing SSB knowledge, policies that affect the behavior beyond individual level factors should also be explored to potentially augment the effects of the SSB tax in populations at high risk of chronic disease.

Another study objective was to understand the relationship between four psychological constructs and taxed beverage intake at baseline and whether this relationship changed after the tax. Of the four constructs analyzed, SSB knowledge and the intention to reduce SSB consumption were significantly associated with taxed beverage intake after the tax was implemented. Participants expressing an intention to reduce SSB intake in the post-tax period were significantly less likely to consume taxed beverages and consumed significantly fewer calories per consumption event than consumers who did not express an intention to change. Intentions are key predictors of behavior according to the Theory of Reasoned Action and Theory of Planned Behavior [48, 49], and a previous meta-analysis found interventions that produced greater intentions to change had greater effects on behavior [50]. In this study, the modification of the tax effect by intentions and SSB knowledge may be due to a signaling effect, whereby the SSB tax not only increases prices but also communicates important information to the consumer about the taxed product, with the justification for the tax made explicit and widely publicized [51, 52].

Although tax awareness, SSB knowledge, and SSB risk perception increased by small amounts after the tax, intention to reduce SSB intake decreased after the tax. According to previous work using the Theory of Planned Behavior framework, attitudes inform intentions, which determine behavior, and intentions may be better predictors of future behavior if they remain stable [53]. In our study, we observed reductions in taxed beverage intake that were greater among those expressing an intention to reduce their SSB intake. However, in the post-tax period, a lower proportion of participants expressed an intention to change. This could be due to the reduced proportion of taxed beverage consumers and the reduced intake among taxed beverage consumers in the post-tax period: by reducing taxed beverage intakes from pre- to post-tax, fewer participants expressed an intention for further reductions. The stability of these intentions may determine the degree to which further reductions in taxed beverage intake follow in the future, and these intentions could also interact with future modifications to the HPL. More work is needed to examine how intentions and intake change over time after SSB taxes, and whether future interventions that support the stability of intentions to change may lead to greater benefits.

The strengths of this study include a large sample size of over 2000 participants at both time periods, allowing sufficient study power to detect changes in beverage intake. Survey coverage of Langa was also extensive, as demonstrated by Supplementary Material 2, increasing our confidence that we effectively sampled the beverage intake of our target population. This is also one of the first studies to examine potential modifiers of an SSB tax among a low income community, an area which is underrepresented in the literature in the context of national SSB taxes. This study builds upon previous work that examined the signaling effect of a SSB tax by measuring associations between self-reported change in beverage intake rather than beverage intake itself [22]. Finally, the present study measures beverage intake with 24-hour recalls, a less biased diet assessment method recommended by the National Cancer Institute for measuring the effects of an intervention on mean dietary intake in a population [54].

This study has several limitations. First, given the pooled cross-sectional nature of our data and pre-post approach, we are unable to make causal claims about the relationships between individual level psychological constructs and participant behavior, and how the SSB tax affected these. Although the majority of policy evaluation studies are observational, future studies on this topic would benefit from linking survey participants across time with their psychological variables and dietary intake. Second, our use of a constant food composition table across time means we are able to track changes in behavior, but we are not capturing any reformulation effects that may have occurred at one-year post tax implementation. This means that some beverages at one-year post tax implementation may be misclassified if the products were reformulated below the 4 g/100mL and should have been classified as untaxed, limiting our ability to completely detect changes in taxed beverage consumption after the tax. We did not examine substitution effects after the HPL as these have been studied elsewhere using purchase data [16] and dietary intake data [17], which found reductions in kcal from taxed beverage were partially compensated by an increase in sugar and energy intake from untaxed beverages, reducing the overall public health impact of the tax. These results may not be generalizable to higher income populations, particularly because these results suggest price may have been a major driver for reductions in SSB intake. Indeed, other research has found the largest changes in sugary beverage intake among the lowest income groups analyzed after an SSB tax in Mexico [26]. The study samples were 65% female in both pre- and post-tax periods, which has some implications for generalizability. On average, females consumed 32 kcal less from taxed beverages in the post-tax period compared to 27 kcal less for males. Therefore, an overrepresentation of females in our study population could mean a slight overestimation of the average effect of the HPL on the Langa population. Finally, there are limitations in our measure of intention to change behavior. The average educational attainment in this study population is low, and surveys were accordingly made to have low complexity, but the simplicity of a binary measure creates a tradeoff that limits its statistical utility.

To our knowledge, this is the first study to test potential behavioral modifiers of an SSB tax using 24-hour dietary recalls, which a more suitable measure for changes in mean population intakes than frequency questionnaires [54] or even cruder intention to change survey responses as an outcome [22]. Further research could build on this approach by collecting more comprehensive data that include measures beyond these four psychological constructs such as media exposure to better understand potential modifiers of behavioral change.

## Conclusion

This study found tax awareness, SSB knowledge, and SSB risk perception increased slightly after the South African SSB tax was implemented in a low-income South African township. Tax awareness remains low, and only SSB knowledge and behavioral intention to change were significantly associated with taxed beverage intake in the post-tax period. The tax effect on SSB intake was modified by SSB knowledge and intention to reduce SSB intake, with higher levels of each associated with greater differences in SSB intake in the post-tax period. Future studies may benefit from longitudinal data collection of more comprehensive psychological and behavioral measures and media exposure to better understand potential drivers of individual-level changes after SSB taxes. However, given this study found a small effect of SSB knowledge and no effect of SSB risk perception, individual level knowledge and beliefs may not be the key drivers of changes in SSB intake for this study population.

## Electronic supplementary material

Below is the link to the electronic supplementary material.

Supplementary Material 1: STROBE-nut_checklist IJBNPA

Supplementary Material 2: Langa Sampling

## Data Availability

Data analyzed for this paper form part of a primary project which is currently being written up in other publications. Select relevant data used for this paper will therefore only be available from the corresponding author upon request and only granted for replication purposes.
