# Supplementary material for: Are intentions to change, policy awareness, or health knowledge related to changes in dietary intake following a sugar-sweetened beverage tax in South Africa? A before-and-after study
Source: Int J Behav Nutr Phys Act. 2022 Oct 28;19:136. doi: 10.1186/s12966-022-01370-5 (PMC9617427; doi:10.1186/s12966-022-01370-5)
Supplement: Supplementary file 1 — Supplementary Material 1: STROBE-nut_checklist IJBNPA [file 12966_2022_1370_MOESM1_ESM.docx]

**Additional File 1. STROBE-nut: An extension of the STROBE statement for nutritional epidemiology**

Lachat C et al. (2016) STrengthening the Reporting of OBservational studies in Epidemiology – Nutritional Epidemiology (STROBE-nut): an extension of the STROBE statement. Plos Medicine 13(6) <http://dx.doi.org/10.1371/journal.pmed.1002036> [pdf](http://journals.plos.org/plosmedicine/article/asset?id=10.1371%2Fjournal.pmed.1002036.PDF) or [online](http://journals.plos.org/plosmedicine/article?id=10.1371/journal.pmed.1002036) version.

| **Item** | **Item nr** | **STROBE recommendations** | **Extension for Nutritional Epidemiology studies (STROBE-nut)** | **Reported on page #** |
| --- | --- | --- | --- | --- |
| **Title and**  **abstract** | 1 | (a) Indicate the study’s design with a commonly used term in the title or the abstract.  (b) Provide in the abstract an informative and balanced summary of what was done and what was found. | **nut-1** State the dietary/nutritional assessment method(s) used in the title, abstract, or keywords. | Title: includes “before-and-after study”; Methods section **Data Sources and Measures:** *Participants* mentions before and after study. Abstract is balanced presentation of what was done and found, including 24-h recalls used to assess diet (nut-1). |
| **Introduction** |  |  |  |  |
| Background rationale | 2 | Explain the scientific background and rationale for the investigation being reported. |  | Introduction: paragraphs 1–4 |
| Objectives | 3 | State specific objectives, including any pre-specified hypotheses. |  | Introduction: paragraph 4 objectives |
| **Methods** |  |  |  |  |
| Study design | 4 | Present key elements of study design early in the paper. |  | Methods:  **Data Sources and Measures** and **Analytical Approach** |
| Settings | 5 | Describe the setting, locations, and relevant dates, including periods of recruitment, exposure, follow-up, and data collection. | **nut-5** Describe any characteristics of the study settings that might affect the dietary intake or nutritional status of the participants, if applicable. | Methods:  sections entitled **Data Sources and Measures** and **Analytical Approach**  Nut-5: N/A |
| Participants | 6 | a) Cohort study—Give the eligibility criteria, and the sources and methods of selection of participants. Describe methods of follow-up.  Case-control study—Give the eligibility criteria, and the sources and methods of case ascertainment and control selection. Give the rationale for the choice of cases and controls.  Cross-sectional study—Give the eligibility criteria, and the sources and methods of selection of participants.  (b) Cohort study—For matched studies, give matching criteria and number of exposed and unexposed.  Case-control study—For matched studies, give matching criteria and the number of controls per case. | **nut-6** Report particular dietary, physiological or nutritional characteristics that were considered when selecting the target population. | **Cross-sectional study:** eligibility, population, and selection described in paragraphs 1-3 of **Data Sources and Measures**.  Nut-6: population selected for being a high consuming of SSBs (paragraph 1 of **Data Sources and Measures:** *Participants***)** |
| Variables | 7 | Clearly define all outcomes, exposures, predictors, potential confounders, and effect modifiers. Give diagnostic criteria, if applicable. | **nut-7.1** Clearly define foods, food groups, nutrients, or other food components.  **nut-7.2** When using dietary patterns or indices, describe the methods to obtain them and their nutritional properties. | Nut-7.1: Classification of taxed and untaxed beverages defined in *Linking dietary data to beverage categories* section of Methods.  Exposures and outcomes defined in *Measuring Psychological Constructs* (including Table 1) and *Main outcome and covariates* of Methods section.  Nut-7.2: N/A |
| Data sources - measurements | 8 | For each variable of interest, give sources of data and details of methods of assessment (measurement).Describe comparability of assessment methods if there is more than one group. | **nut-8.1** Describe the dietary assessment method(s), e.g., portion size estimation, number of days and items recorded, how it was developed and administered, and how quality was assured. Report if and how supplement intake was assessed.  **nut-8.2** Describe and justify food composition data used. Explain the procedure to match food composition with consumption data. Describe the use of conversion factors, if applicable.  **nut-8.3** Describe the nutrient requirements, recommendations, or dietary guidelines and the evaluation approach used to compare intake with the dietary reference values, if applicable.  **nut-8.4** When using nutritional biomarkers, additionally use the STROBE Extension for Molecular Epidemiology (STROBE-ME). Report the type of biomarkers used and their usefulness as dietary exposure markers.  **nut-8.5** Describe the assessment of nondietary data (e.g., nutritional status and influencing factors) and timing of the assessment of these variables in relation to dietary assessment.  **nut-8.6** Report on the validity of the dietary or nutritional assessment methods and any internal or external validation used in the study, if applicable. | Nut-8.1: use of 24-hour dietary recalls described in *Measuring dietary intake* section of Methods.  Nut-8.2: food composition data used and linking of dietary intake data to nutrient data described in *Linking dietary data to beverage categories* section of Methods.  Nut-8.3: N/A  Nut-8.4: N/A  Nut-8.5: to assess influencing factors of diet, this study was analyzing potential modifiers of the effect of an SSB tax on dietary intake. Covariates that influence intake were also controlled for.  Nut-8.6: 24-hour dietary recalls collected using a multiple pass method are widely accepted method for evaluating the effects of an intervention. Citation 54 is referenced in the Discussion. |
| Bias | 9 | Describe any efforts to address potential sources of bias. | **nut-9** Report how bias in dietary or nutritional assessment was addressed, e.g., misreporting, changes in habits as a result of being measured, or data imputation from other sources | Nut-9: Methods **Analytical approach** (Adjusted analysis using two-part model). Covariate adjustment and assessing potential bias due to LSM groups included are described in *Main outcome and covariates* section of Methods. |
| Study Size | 10 | Explain how the study size was arrived at. |  | Paragraphs 1-4 of *Participants* section of Methods. |
| Quantitative variables | 11 | Explain how quantitative variables were handled in the analyses. If applicable, describe which groupings were chosen and why. | **nut-11** Explain categorization of dietary/nutritional data (e.g., use of N-tiles and handling of nonconsumers) and the choice of reference category, if applicable. | Nut-11: No exclusion or re-categorization of consumers/non-consumers of taxed beverages as the key outcome was mean SSB intake in the population: the key target of the SSB tax. The two-part model better accounts for non-consumers compared to OLS regression. |
| Statistical  Methods | 12 | (a) Describe all statistical methods, including those used to control for confounding  (b) Describe any methods used to examine subgroups and interactions.  (c) Explain how missing data were addressed.  (d) Cohort study—If applicable, explain how loss to follow-up was addressed.  Case-control study—If applicable, explain how matching of cases and controls was addressed.  Cross-sectional study—If applicable, describe analytical methods taking account of sampling strategy.  (e) Describe any sensitivity analyses. | **nut-12.1** Describe any statistical method used to combine dietary or nutritional data, if applicable.  **nut-12.2** Describe and justify the method for energy adjustments, intake modeling, and use of weighting factors, if applicable.  **nut-12.3** Report any adjustments for measurement error, i.e,. from a validity or calibration study. | **nut-12.1**: All statistical methods are described in the **Analytical Approach** section of the Methods.  **nut-12.2**: References 33 and 36 are used to justify the selection of the two-part model for dietary intake. |
| **Results** |  |  |  |  |
| Participants | 13 | (a) Report the numbers of individuals at each stage of the study—e.g., numbers potentially eligible, examined for eligibility, confirmed eligible, included in the study, completing follow-up, and analyzed.  (b) Give reasons for non-participation at each stage.  (c) Consider use of a flow diagram. | **nut-13** Report the number of individuals excluded based on missing, incomplete or implausible dietary/nutritional data. | Nut-13: The number of individuals at each stage of study, and the process of excluding individuals with implausibly low intakes, are discussed in the 4^th^ paragraph of **Data Sources and Measures** in the Methods. Reasons are given for exclusion at each stage. |
| Descriptive data | 14 | (a) Give characteristics of study participants (e.g., demographic, clinical, social) and information on exposures and potential confounders  (b) Indicate the number of participants with missing data for each variable of interest  (c) Cohort study—Summarize follow-up time (e.g., average and total amount) | **nut-14** Give the distribution of participant characteristics across the exposure variables if applicable. Specify if food consumption of total population or consumers only were used to obtain results. | Nut-14: Tables 2 of Results section. Consumers/non-consumers are dealt with in the modelling, not classification, as described in Analytical Approach. |
| Outcome data | 15 | Cohort study—Report numbers of outcome events or summary measures over time.  Case-control study—Report numbers in each exposure category, or summary measures of exposure.  Cross-sectional study—Report numbers of outcome events or summary measures. |  | Table 3 of Results section. |
| Main results | 16 | (a) Give unadjusted estimates and, if applicable, confounder-adjusted estimates and their precision (e.g., 95% confidence interval).  Make clear which confounders were adjusted for and why they were included.  (b) Report category boundaries when continuous variables were categorized.  (c) If relevant, consider translating estimates of relative risk into absolute risk for a meaningful time period. | **nut-16** Specify if nutrient intakes are reported with or without inclusion of dietary supplement intake, if applicable. | (a) Confounder adjusted estimates are presented in Table 4, including 95% Confidence Intervals.  nut-16: N/A  (b) Estimations at representative levels (every 10 percentage point increases for risk perception and knowledge) are presented in Table 4.   (c)Absolute measures are reported to allow for clearer interpretation of public health impact. |
| Other analyses | 17 | Report other analyses done—e.g., analyses of subgroups and interactions and sensitivity analyses. | **nut-17** Report any sensitivity analysis (e.g., exclusion of misreporters or outliers) and data imputation, if applicable. | Interaction results described in section 3 of Regression results. |
| **Discussion** |  |  |  |  |
| Key results | 18 | Summarize key results with reference to study objectives. |  | Paragraph 1 of Discussion. |
| Limitation | 19 | Discuss limitations of the study, taking into account sources of potential bias or imprecision. Discuss both direction and magnitude of any potential bias. | **nut-19** Describe the main limitations of the data sources and assessment methods used and implications for the interpretation of the findings. | Limitations described in paragraph 8 of Discussion, including methods limitations. |
| Interpretation | 20 | Give a cautious overall interpretation of results considering objectives, limitations, multiplicity of analyses, results from similar studies, and other relevant evidence. | **nut-20** Report the nutritional relevance of the findings, given the complexity of diet or nutrition as an exposure. | Findings are not overstated. We make clear that changes in potential modifiers were small and probably did not have a large modifying of the tax effect on taxed beverage intake. |
| Generalizability | 21 | Discuss the generalizability (external validity) of the study results. |  | Generalizability mentioned in paragraph 8 of Discussion. |
| **Other information** |  |  |  |  |
| Funding | 22 | Give the source of funding and the role of the funders for the present study and, if applicable, for the original study on which the present article is based. |  | Funding sources given in the **Declarations** section. |
| *Ethics* |  |  | **nut-22.1** Describe the procedure for consent and study approval from ethics committee(s). | Ethics approval and consent described in **Ethics approval and consent to participate** section of **Declarations** section. |
| *Supplementary material* |  |  | **nut-22.2** Provide data collection tools and data as online material or explain how they can be accessed. | 24-hour recall is a standard method and the multiple pass method was used by trained nutritionists, which is not captured on the 24-hour dietary intake form. |
