# Supplementary material for: Are intentions to change, policy awareness, or health knowledge related to changes in dietary intake following a sugar-sweetened beverage tax in South Africa? A before-and-after study
Source: Int J Behav Nutr Phys Act. 2022 Oct 28;19:136. doi: 10.1186/s12966-022-01370-5 (PMC9617427; doi:10.1186/s12966-022-01370-5)
Supplement: Supplementary file 2 — Supplementary Material 2: Langa Sampling [file 12966_2022_1370_MOESM2_ESM.docx]

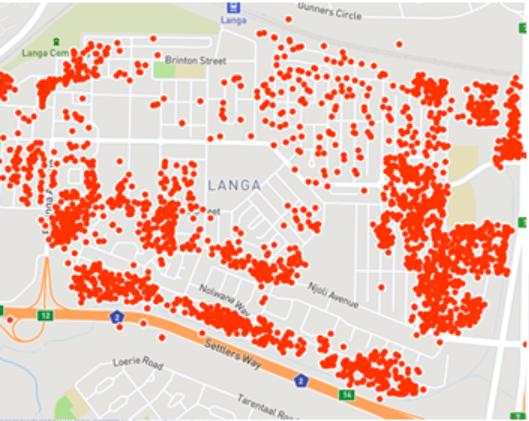


**Additional File 2.** Sampling map for Langa township created using geocodes from 24-hour diet recalls.
